# Teacher’s health and presenteeism

**DOI:** 10.47626/1679-4435-2022-973

**Published:** 2024-09-24

**Authors:** Tatiana Varejão Garcia, Carmen Casquel Juliani

**Affiliations:** 1 Public Health, Universidade Estadual Paulista “Júlio de Mesquita Filho”. Botucatu, SP Brazil

**Keywords:** school teachers, presenteeism, occupational health, docentes, presenteísmo, saúde do trabalhador

## Abstract

Presenteeism is a relatively new phenomenon affecting the health of employees, and studies on this topic are scarce. It refers to being physically present in the workplace but mentally or physically unable to fully perform one’s duties. For teachers in particular, this is influenced by internal and external factors that determine their relationship with work. The objective of this study was to understand how presenteeism developed among teachers from a school in the city of São Paulo, Brazil, which offers secondary education programs including basic professional and technical qualification. This was a qualitative study using Bardin’s content analysis method. Twenty teachers suffering from presenteeism were randomly selected to compose the purposive sample. Data were collected from September to December 2019. Participants complaints were mainly related to school organization and their own relationship with work. Several factors lead to illness and impact teachers’ health and quality of life, such as physical and emotional exhaustion, difficulties in recovering health, suffering, underperformance, difficulty taking sick leaves, and professional and financial damages. Presenteeism involves personal, financial, and sociocultural issues, as well as organizational factors that make teachers feel obligated to work even when they are sick to perform their duties.

## Abstract (pt)

O presenteísmo é um fenômeno relativamente novo que afeta a saúde dos trabalhadores. Estudos sobre o tema são escassos. O presenteísmo refere-se a estar fisicamente presente no local de trabalho, mas mental ou fisicamente incapaz de desempenhar plenamente suas funções. Para os professores, isso é influenciado por fatores internos e externos que determinam a sua relação com o trabalho. O objetivo deste estudo foi compreender como o presenteísmo se desenvolveu entre professores de uma escola pública de ensino médio e técnico na cidade de São Paulo. Trata-se de um estudo qualitativo que utilizou o método de análise de conteúdo de Bardin. Foram selecionados aleatoriamente 20 professores com presenteísmo para compor a amostra proposital. A coleta de dados foi realizada no período de setembro a dezembro de 2019. Questões relacionadas à organização do trabalho e à relação do indivíduo com o trabalho propriamente dito foram, entre outras, as que mais apareceram na pesquisa. Diversos fatores levam ao adoecimento e impactam a saúde e a qualidade de vida dos professores, tais como desgaste físico e emocional, dificuldades na recuperação da saúde, sofrimento, queda no rendimento, dificuldade de afastamento e prejuízos profissionais e financeiros. O presenteísmo envolve questões pessoais, financeiras e socioculturais, além de fatores organizacionais que levam o professor a trabalhar sem condições de saúde para cumprir suas funções no trabalho.

## INTRODUCTION

As in any other occupational activity, teaching involves situations that may lead to absenteeism and presenteeism, which are directly linked to the health-disease process.

Both presenteeism and absenteeism due to work-related illness may act as indicators of poor well-being and health, but presenteeism in particular is an indicator of productivity loss associated with physical and mental health and can only be reported by the employees themselves.^1^ In addition, there is also the phenomenon of *leaveism,* which is the practice of employees using allocated time off such as annual leave entitlements, flexi hours banked, and re-rostered rest days to take time off when they are in fact unwell; employees taking work home that cannot be completed in normal working hours; and employees working while on leave or holiday to catch up.^2^

Presenteeism refers to being physically present but mentally or physically unable to fully perform one’s duties, which is akin to being at work but not fully engaged.^3^ Contrary to absenteeism, investigating presenteeism is challenging because its manifestation is not always clear. It should be noted that presenteeism is not simply the opposite of absenteeism. It significantly impacts work performance, potentially reduces productivity, and has serious consequences for both the individual and the work setting.^4^

Contrary to absenteeism, presenteeism is not always apparent. While it is easy to know when someone is physically absent from work, it is often unclear when – or how much – an illness or medical condition impairs the performance of someone who is physically present at the workplace without exhibiting any outward signs or symptoms.^5^

Therefore, knowledge of teachers’ health conditions is crucial from a social and personal perspective as well as from an organizational one. The author’s experience as a teacher and school administrator motivated us to investigate how presenteeism leads to absenteeism and directly affects education professionals. This study aimed to reflect and contribute to the advancement of knowledge about teacher’s health conditions and presenteeism to guide teaching practices and management.

The occupational environment is a space where human relations are influenced by personal, social, and cultural factors.^6^ The role of the teacher in current society is simultaneously stressful and challenging, which has been gradually turning teaching into a profession with physical and mental risks.^7^

An analysis of teaching activities reveals a significant intensification of labor, which is related to job instability, involving economic factors (salary, working time, employment contracts) and working conditions (changes in job structures and production processes with the use of new tools and flexible management models) that alter work routines and forms of control.^8^

Among the countless stressors found in the occupational setting are excessive workload, job instability, increasingly demanding job markets, family conflicts, social and financial problems, interpersonal relationships, and the general conditions to which employees are exposed daily, which directly affect their cognitive, physical, and emotional capacities. Consequently, employees develop illnesses that are often ignored, and neglect of clinical symptoms often leads to prolonged sick leave, presenteeism and absenteeism. For teachers in particular, the aforementioned stressors are exacerbated by demands relating to their professional and personal performance, threatening their well-being and self-esteem.^7^

According to Souza et al.,^9^ until the 1960s, most education professionals enjoyed some job security, stable employment, and a certain social prestige. From the 1970s onwards, the increase in the population’s demands for social protection led to the growth of civil service and free public services, including education. A greater and often unattainable burden was then transferred to teachers, such as the responsibility to fill gaps, manage overcrowded classrooms, and deal with different socioeconomic and cultural levels, among others.

Since the 1990s, changes in education policies have led schools, whether private or public, to be seen as a business, following neoliberalism principles. Teaching roles evolved into those of a “versatile” teacher who is responsible for the success or failure of education.^7^ Pressures and demands increased, destabilizing teachers, as the focus shifted to results (expressed in grades), to which students and families began to pay more attention, while the teaching-learning process was sidelined.^10^ Simultaneously, a method of “continuous progression” was established in all schools in the state of São Paulo, Brazil, including high school and technical education.

Souza et al.^9^ stated that “faced with the increasing instability and deterioration of health conditions at work, investigations into how the health-illness-work process affected education professionals in Brazil and other countries began in the 1990s.” Schools are spaces that can generate suffering and stress due to issues such as work devaluation, low income, personal problems, authoritarian management, and blame for negative outcomes, among others.

Health-related issues remained marginalized in the education sector, whether by school management, labor movements, or by teachers themselves, who, accustomed to caring for others, struggle to focus on themselves, their well-being, and their health. Signs of illness are overlooked or minimized and are only recognized after they become serious.^8^

According to Gomes et al.,^11^ despite stress occurring in daily life, it becomes more significant when associated with work. Given the demands and activities that teachers undertake, they often find themselves in situations for which they do not feel prepared, whether due to their professional training or previous experiences. The new demands brought about by the most recent policies require teachers to develop new skills necessary for the full exercise of their activities. The system expects teachers to be prepared, have proper training, and be motivated to fully master the classroom and meet the different school needs with the urgency they require.^12^

The effect of these demands on health is not always apparent. Workers develop mechanisms to alter work goals or methods. When subjected to embarrassing situations, the expected results are achieved through inner changes, causing both short-and long-term health impairment, with immediate or future changes.^13^ Teachers ultimately succumb to presenteeism, causing an increase in workload and the exacerbation or emergence of illnesses.

Santana et al.^14^ observed that teachers become ill as a result of several work-related attributes, ultimately needing to take time off. Among the conditions that most affect teachers are musculoskeletal disorders, which increase absenteeism rates, causing losses to the school, students, education, and the teachers themselves, as well as costs related to treatment and social security benefits. Voice disorders also contribute to absenteeism and are associated with environmental factors such as excessive workload and poor working conditions, as well as psychosocial factors. The noisy environment of a classroom is another determining factor for absenteeism.^15^

Studies show that not all teachers take time off work. To fulfill their professional responsibilities, teachers avoid missing work based on social and ethical beliefs, even if they are not in ideal physical condition.^15^

### OBJECTIVES

This study aimed to understand the phenomenon of presenteeism among teachers who have experienced it.

## METHODS

This was a qualitative study using the content analysis method,^16^ which involves studying both the explicit content and implicit messages conveyed through language, pauses, and nuances.

The study setting was a school in the city of São Paulo, Brazil, which offers secondary education programs including basic professional and technical qualification in administration, electrotechnics, fashion, visual communication, as well as modular courses in administration, electronics, visual communication, and occupational safety.

Data were collected from September to December 2019. At the time of data collection, the school had approximately 1,230 students and 124 active teachers, which constituted the study population. Classes were administered in the morning, afternoon, and evening for both secondary education and modular courses. Purposive sampling included 20 randomly invited teachers, of whom 45% were men and 55% were women, with a mean age of 43 years and length of service of 14 years.

Interviews were conducted until the research objectives were met and theoretical saturation was achieved. The inclusion criterion was having experienced presenteeism, determined by a previous survey. Following this criterion, the study sample was randomly composed according to the scheduling availability of both researcher and participant for the interview.

To understand presenteeism among teachers, after informed consent was obtained, individual interviews based on a script with open-ended questions were conducted by the researcher at predetermined locations and times. A private room at the school was designated for the interviews, which were recorded and lasted an average of 5 minutes.

Data analysis allowed us to understand the phenomenon of presenteeism. Topics and information were discussed that led to reflections on the daily routine of teachers within the school environment, individual profiles, and cultural issues related to work. This study was approved by the ethics committee under number 3.524.721.

## RESULTS

Participants reported that there are no substitute teachers available to take over classrooms if they need to be absent for any reason, requiring them to work even when they are in poor health. This causes distress on both the teacher and the entire school staff. “*The system places us as irreplaceable, so if you’re not there, there’s no one to replace you” “You can’t miss [work] because there’s no one to replace you in the classroom, and if you’re absent, it’s an operational problem.”*

Araújo^3^ observes that the ease or difficulty of replacing employees, including teachers, affects the rates of presenteeism. If a worker feels irreplaceable, they are more likely to work when sick.

With regard to sick leave, participants reported that when a doctor grants them sick leave for the day or for at least a few hours, it is still not enough, as some conditions require more time for an effective recovery. Some health issues cannot be resolved without adequate time off. Voice disorders (aphonia) may develop due to overcrowded classrooms, where teachers need to strain their voice to be heard. Because of their commitment to colleagues and students, teachers end up working under the worst health conditions. “*The doctor didn’t grant me sick leave even though I lost my voice. What illness was I suffering from? But I can’t work because my voice is my instrument, but teachers can suffer from loss of voice,” “Because we only go to the doctor after our workday, it doesn’t really matter if the doctor grants us sick leave or not.”*

Penteado & Souza Neto^17^ emphasize that issues related to the body, health, and life should be integrated into the teaching culture to promote the appreciation and quality of education. Teachers only seek medical help when they are at the limit of their strength, when the discomfort becomes unbearable, and other defense mechanisms no longer work. By playing the role of “caregiver” for students, teachers often do not worry about themselves. There is a lack of health care and promotion in the school setting for teachers, as well as a neglect of their well-being. Voice disorders may lead to illness, reduced work capacity, underperformance, and absenteeism and affect peer relationships, causing social, economic, professional, and personal damage.^18^

Teachers lack support from the school staff/management/students when their absence generates work overload and cannot be covered by those who are present at school, whether from the administrative, management, or teaching departments. Being aware of this, teachers avoid missing work even when their health is compromised. Some teachers reported feeling pressured by the school to “*Even when I don’t teach, I can tell that the school board and management were relieved that I showed up, even though I was aphonic,” “We have to deal with the pressure that the school puts on us.”*

According to Araújo,^3^ a study conducted in France showed that the example set by the management department is one of the main causes of presenteeism, that is, the behavior of a manager (director, coordinator) can influence a team regarding work attendance.

Teachers also reported that the responsibility and importance attributed to their work and the respect they have for students, the school staff, and the school itself leads to both physical and emotional exhaustion. Although they are committed to being present in class and to provide education for students, in addition to respecting colleagues and students, their efforts are often not recognized by some colleagues and the school management. “*And although I was in pain, I still taught the class, because I didn’t want to let the students down”, “I attend mainly out of respect for the students, because I think that, if a student is absent, the class will still happen; but if the teacher is absent, it will affect all students.”*

Commitment to work is one of the main factors related to presenteeism, as the individual creates bonds and makes commitments with people, groups, or institutions.^19^ Shimabuku et al.^20^ concluded that presenteeism results from the perception that one’s absence could lead to increased workload for their colleagues or themselves. Presenteeism is characterized by loyalty, motivation, and a sense of responsibility.

Underperformance occurs when a teacher is at school but should be at home taking care of their health. The teacher is aware that their performance in class will not be the best but feels obligated to be present even if the expectations they have on a normal day are not met. “*You are not up to the task at the moment and end up stalling,” “Working when you’re not well, sick, or in pain makes you underperform, because you can’t perform well when you’re not physically well, but you still have to be present.”*

Work overload and fatigue of body and mind, combined with working conditions and daily stress in the classroom, impair work performance and the quality of classes.^21^ Teachers report that working when sick is common, including physical problems, such as sore throat, aphonia, migraine, rhinitis, back pain, fractures, and heart disease, as well as emotional problems related to family and stress. “*I have serious cervical spine problems (...) and neck pain, constant stiff neck, waking up in pain, but I go to work anyway,” “I also have migraines (...) I take painkillers and continue working to accomplish what I planned for the day even though I’m in pain.”*

Johns^22^ states that employees who go to work even when they feel sick are usually suffering from an acute, episodic, or chronic illness (such as the flu, migraine, or diabetes, respectively), as more serious health problems require the worker to be absent from work, usually for longer periods, and therefore does not constitute presenteeism.

In Paschoalino’s study,^23^ teachers in the public sector suffering from presenteeism, although not at risk of losing their jobs for being sick, maintain teaching activities to feel useful and accomplish their mission of providing education. Teachers strongly believe that their profession is one of dedication, in which you think about the student and put your own needs aside.

Regarding cultural values, participants said that they do not feel comfortable missing work even if they need care, that they feel they are putting students and colleagues in uncomfortable positions, and that many people do not like when teachers miss work despite it being their right. Some participants said they only miss work when movement or communication is impossible. Teachers do not know how to set a limit for themselves, meaning they do not know when to stop. “It’s *funny, I shouldn’t feel bad, but I feel bad about missing work,” “There is something ingrained in our culture that a good employee is one who doesn’t miss work, works even when sick, and fulfills all their obligations.”*

The teacher’s identity is built from childhood roots (inherited social identities), the work done throughout the school journey (learning from observation and experience), the process of professional socialization, and occupational socialization (professional practices). It is the result of a biographical and relational process.^24^

For Johns,^22^ some professions related to health care, such as doctors and nurses, and others in the education sector, such as teachers and caregivers, are more prone to presenteeism, suggesting a culture based in part on loyalty and concern for vulnerable clients, namely the sick and children.

## CONCLUSIONS

The statements related to presenteeism demonstrate various factors that make teachers ill and impact their health and quality of life, such as physical and emotional exhaustion, difficulties in health recovery, suffering, underperformance, difficulty taking time off, professional and financial losses, lack of support from school management, teaching staff, and students, as well as the lack of substitute teachers.

In the interviews, factors such as responsibility, commitment, and cultural values stood out as relevant and significant, demonstrating how teachers almost spontaneously give themselves to their work, without being able to set boundaries to preserve their health. Teachers justify, in many ways, the importance of their presence at school, without concern for their physical and emotional well-being. Guilt, self-pressure, and difficulty respecting one’s limits, recognizing illness, and internal conflicts are determining factors for teachers to show up for work even when they are not in good health.

Presenteeism does not always lead to absenteeism, which indicates that an illness may be present or developing without being adequately recognized and treated. Presenteeism is a silent phenomenon that, when it comes to teachers who have difficulty setting limits and identifying when they are sick, whether because they overlook signs of illness or because they do not value prevention and promotion of their own health, becomes more challenging. When the phenomenon of presenteeism cannot be quantified, teachers become ill without anyone noticing.

Teachers are involved in many school campaigns about the health and well-being of the school community, including students and families, but their own health is left aside.

Our findings contribute to the discussion about the boundaries between presenteeism and absenteeism and the health of those who, focused on promoting the health of others, put their own health in second place. Our results can help support educational actions to improve the quality of life of teachers in the workplace.

We should all reflect on how much attention is being paid to teachers’ health, how many public health actions are directed at teachers, and how public policies could contribute in this regard, as is the case in other sectors of occupational health.
